# Toward a Neuroscience of Adult Cognitive Developmental Theory

**DOI:** 10.3389/fnins.2018.00004

**Published:** 2018-01-23

**Authors:** Fady Girgis, Darrin J. Lee, Amir Goodarzi, Jochen Ditterich

**Affiliations:** ^1^Department of Neurosurgery, University of California, Davis, Davis, CA, United States; ^2^Department of Neurosurgery, University of Toronto, Toronto, ON, Canada; ^3^Center for Neuroscience, University of California, Davis, Davis, CA, United States; ^4^Department of Neurobiology, Physiology and Behavior, University of California, Davis, Davis, CA, United States

**Keywords:** cognitive developmental theory, self-related processing, theory of mind, Dynamic Field Theory, Research Domain Criteria

## Abstract

Piaget's genetic epistemology has provided the constructivist approach upon which child developmental theories were founded, in that infants are thought to progress through distinct cognitive stages until they reach maturity in their early 20's. However, it is now well established that cognition continues to develop after early adulthood, and several “neo-Piagetian” theories have emerged in an attempt to better characterize *adult* cognitive development. For example, Kegan's Constructive Developmental Theory (CDT) argues that the thought processes used by adults to construct their reality change over time, and reaching higher stages of cognitive development entails becoming objectively aware of emotions and beliefs that were previously in the realm of the subconscious. In recent years, neuroscience has shown a growing interest in the biological substrates and neural mechanisms encompassing adult cognitive development, because psychological and psychiatric disorders can arise from deficiencies therein. In this article, we will use Kegan's CDT as a framework to discuss adult cognitive development in relation to closely correlated existing constructs underlying social processing, such as the perception of self and others. We will review the functional imaging and electrophysiologic evidence behind two key concepts relating to these posited developmental changes. These include self-related processing, a field that distinguishes between having conscious experiences (“being a self”) and being aware of oneself having conscious experiences (“being aware of being a self”); and theory of mind, which is the objective awareness of possessing mental states such as beliefs and desires (i.e., having a “mind”) and the understanding that others possess mental states that can be different from one's own. We shall see that cortical midline structures, including the medial prefrontal cortex and cingulate gyrus, as well as the temporal lobe, are associated with psychological tasks that test these models. In addition, we will review computational modeling approaches to cognitive development, and show how mathematical modeling can provide insights into how sometimes continuous changes in the neural processing substrate can give rise to relatively discrete developmental stages. Because deficiencies in adult cognitive development can result in disorders such as autism and depression, bridging the gaps between developmental psychology, neuroscience, and modeling has potential implications for clinical practice. As neuromodulation techniques such as deep brain and transcranial stimulation continue to advance, interfacing with these systems may lead to the emergence of novel investigational methods and therapeutic strategies in adults suffering from developmental disorders.

## Introduction

The field of cognitive development was pioneered and largely developed by Jean Piaget in the early to mid 1900's. His approach to genetic epistemology, or study of the origins of knowledge, took a constructivist stance, in that he focused on how children used their ideas and social experiences to construct their reality over time. He defined several stages of development, of which the final stage was thought to conclude around the 25th year of life (Piaget, 1971). However, many adults continue their cognitive development after their mid 20's, and as a result several theories have emerged to better characterize stages of *adult* cognitive development (Fischer, 1980; Basseches, 1984; Wilber, 2006). The psychologist Robert Kegan developed and empirically tested one of the more prominent of these theories, termed Cognitive Developmental Theory or CDT (Kegan, 1994; Kegan and Lahey, 2009).

CDT and other developmental theories deal with the nature of adult thought, the way in which it develops, and its implications in everyday life. As a result, the applications of these theories are numerous and span many domains, including business, education, spirituality, and medicine. Progressing through the described cognitive stages, of which there are five in the case of CDT, implies reaching higher levels of cognitive development. In doing so, individuals obtain novel perspectives and a dialectical manner of thinking, allowing them to approach ambiguity and conflict in more constructive and altruistic ways, both internally and in their interactions with others. In a related fashion, it can be theorized that deficiencies in these perspectives and techniques, or in other words a lack of advancement through the developmental stages, can result in a more self-centered and narrow worldview, an inability to deal with conflict, psychological distress, and in some cases the occurrence of psychiatric disease.

Despite the pervasiveness of these theories and commonality of their ideas, they have largely remained in the realm of the social sciences, namely psychology, sociology, and education. In order for them to permeate the neurosciences, however, investigations into these adult developmental concepts need to be transitioned from psychological methods to those of neural imaging, neurophysiology, and computational neural modeling. In essence, we pose the question: Could the cognitive developmental stages of Kegan be characterized by neurophysiologic states and neural network activity, and could neuroscientific methods be used to identify deficiencies therein? Answering this question would be the first step toward finding methods to interface with these brain systems, both to help decode the underlying processes and eventually to attempt to modify pathological neural activity using neuroprosthetic devices.

While it is currently not possible to answer this question, as there are no dedicated imaging or electrophysiologic studies directly targeting adult cognitive developmental theory thus far, we will work toward establishing a framework in which to explore various hypotheses. First, using the proposition that several aspects of neural development mirror and coincide with cognitive development (Gu et al., 2015), we'll look at structural changes in brain regions and connections in the progression from adolescence into adulthood. Next, we'll introduce Kegan's CDT, using a hypothetical example to better demonstrate its ideas, while exploring parallel concepts of the Research Domain Criteria. Then, we'll attempt to gain neuroscientific insights into CDT by examining the related concepts of self-related processing and theory of mind. Finally, we will have a look at how mathematical modeling shows promise in capturing and predicting developing neural networks.

## Changes in structural brain networks

Human cognition is composed of large-scale networks that consist of brain regions that are functionally coherent at rest and collectively active during cognitive tasks (Dosenbach et al., 2007; Power et al., 2011). These brain networks comprise a collection of neurons or brain areas and their connections, of which there are two general types: structural and functional. Structural connectivity entails the physical connection between different regions in the brain, while functional connectivity is the correlation or coherence of information between various regions of the brain over time. In addition, structural connectivity is believed to exert a causal influence over functional connectivity (Betzel et al., 2014), in that brain regions require a physical connection before they can work toward a common function.

Over the course of development through childhood to adolescence and eventually into adulthood, there are many changes in the organization of the structural brain network. Longitudinal MRI studies have shown that as children grow toward adolescence, parietal and frontal cortices thin and the temporal cortex thickens. There is also an increase in the overall number of neural nodes and white matter density during childhood, indicating increased connectivity, while they remain relatively stable during adolescence (Betzel et al., 2014). Furthermore, there are changes in structural connectivity within specific brain regions, as demonstrated elegantly using graph theory. In an analysis of over 200 normally growing children and adolescents (Khundrakpam et al., 2013), primary sensorimotor connectivity decreased with age, while paralimbic and associative connectivity increased with age. More specifically, it was found that subjects in late childhood showed a relative reduction in the efficiency of local connectivity while there was an increased global efficiency. Potentially this may indicate that there is a time window of brain plasticity occurring during late childhood, one that may accommodate new developmental tasks faced during adolescence.

In addition to structural connectivity changes, there are also functional network connectivity changes throughout development into adulthood, as described in imaging and EEG studies (Anokhin et al., 2000). fMRI studies show that there is an increase in network modularity and functional connectivity during adolescence and young adulthood (Marek et al., 2015), and a decrease over the course of older adulthood (Betzel et al., 2014; Cao et al., 2014). This, as evidenced by decreased white matter connectivity, is hypothesized to account for cognitive decline in later years (O'Sullivan et al., 2001). It is also possible that “developmental miswiring,” or deficiencies and abnormalities in functional connectivity during childhood, may account for developmental disorders and neuropsychological conditions later in life (Di Martino et al., 2014). For example, there is speculation that the decrease in delta oscillations during adolescence, as measured by EEG during sleep, may affect frontal cortex maturation and lead to the onset of schizophrenia (Feinberg and Campbell, 2010).

Similar to and in relation to network connections in development, many cognitive processes are thought to be associated with specific cortical networks. The most commonly studied networks include the default-mode, attention, salience, cognitive control, motor, and auditory networks. While primary motor and sensory systems exhibit a high degree of separation and limited connections to other modules, high order cognitive networks have more between-module connectivity (Power et al., 2011). As can be expected, these cognitive modules undergo significant changes throughout youth (Power et al., 2010; Satterthwaite et al., 2013) and adulthood (Fair et al., 2009; Kelly et al., 2009; Supekar et al., 2009; Tian and Ma, 2017).

The default mode network (more specifically the posterior cingulate cortex, medial prefrontal cortex, precuneus, insula) has been shown to be associated with progressively diminishing posterior attention networks throughout adulthood, with anterior to posterior as well as long-range connections disrupted most severely (Andrews-Hanna et al., 2007; Koch et al., 2010; Tomasi and Volkow, 2012). It has been suggested that the decreased functional connectivity throughout aging (Damoiseaux et al., 2008) has been correlated with the structural network changes in the default mode network (Liu et al., 2017). While these changes could be due to network decline, the structural and functional changes alternatively could be due to increased efficiency in these networks. However, the inability to suppress the default mode network as an individual ages has been associated with decreased performance on cognitive tasks (Persson et al., 2006; Damoiseaux et al., 2008). Concomitantly, there is an increase in frontal and parietal activity with aging, which is hypothesized to be an inability to shift out of the default network to active cognitive processing (Park and Reuter-Lorenz, 2009; Mowinckel et al., 2012). This may be evidence that as an individual becomes older, they use different neural networks for cognitive tasks, such as conflict resolution (Salami et al., 2014).

Although several cognitive networks show decline into later adulthood, some can continue to develop and grow, such as task-positive networks (Mowinckel et al., 2012). Cognitive control and salience networks have also been shown via fMRI to continue to increase into adulthood, in that functional changes in dorsal anterior cingulate cortex associated with error regulation increased in correlation with increasing age (Velanova et al., 2008). In addition, networks associated with theory of mind and self-related processing are also associated with development, as will be explored in later sections. But, prior to exploring these concepts, we'll introduce and discuss CDT, as many of its ideas relate and apply to these cognitive processes.

## Kegan's cognitive developmental theory (CDT)

Kegan's theory joins two separate philosophies, those of constructivism and developmentalism. Constructivists argue that individuals create meaning through their experiences rather than use their experiences to discover a pre-constructed meaning. In contrast, developmentalists argue that individuals cognitively progress and mature over the course of their lives. Constructive-developmentalists, therefore, believe that the systems people use to make meaning in their lives change and develop over time. Kegan (1994) adds that this growth is possible when one is able to reflect upon something in a fresh light and subsequently come to understand it in a different way, that is to take something that was once Subject and make it Object.

That which is Subject includes emotions, assumptions, beliefs, and the other ways in which people create meaning in their lives that are hidden within the subconscious. As a result, people are unknowingly shaped by and are unable to objectify these elements. In contrast, that which is Object are those elements currently available to our conscious mind, and as such, we are aware of them, able to reflect on them, and able to be in control of them (Kegan, 1994; Solms, 2014). Kegan holds that moving an element from Subject to Object, that is, objectifying something to which one was previously subject, fuels cognitive development, in that the more one takes as Object, the more one can appreciate and understand, and the more complex one's overall outlook.

As someone's meaning-making evolves, so too do they progress through Kegan's five Orders of Mind, consecutive stages that indicate the complexity with which individuals construct their reality (Kegan, 1994). Different orders of mind represent different levels of cognitive development, and therefore people in the different orders have different ways of viewing the world and dealing with conflict and ambiguity. While five orders or stages have been described, it is important to note that the majority of adults live in the third stage, some eventually reach the fourth stage, and only a handful ever attain the fifth and ultimate stage (Kegan and Lahey, 2009). To demonstrate these orders of mind and their approach to dealing with conflict, we shall focus on stages three to five using politics as an example, and examine how a hypothetical person from each order of mind of a particular political party would construct their reality and deal with ideas and people of other political parties.

Individuals in Kegan's third stage of cognitive development possess a “socialized mind” (Kegan and Lahey, 2009, p. 17), meaning they shape their identities based on the guiding principles present in their personal environments, principles defined by people and institutions they hold in high esteem. In the case of our hypothetical political person, this environment would constitute their particular political party and its leaders, as well as personal acquaintances such as family and friends who share the same political attitudes. This third order person would therefore construct his political identity in order to cohere with the ideas, beliefs, and guidelines delineated by these people and the party. He would likely denounce other political views as incorrect, and avoid inter-political dialog altogether, except when undertaken with the aim of converting others to his own views.

A political person at the fourth order of mind is capable of the same thought processes as one at the third order, but in addition to this capability, also possesses a mentality that is more complex compared to her third-order counterpart. This is because she is able to “self-author” (Kegan and Lahey, 2009, p. 17) her own set of political beliefs, rather than rely on a higher institution to define these beliefs for her. This means that she has a political identity that is separate from that of the party, and as a result is no longer held captive by the opinions of that institution because those opinions have now become Object to her. Someone at the fourth order, by definition, is able to step far enough back from their surroundings, in this case the beliefs of their political community, to generate a personal authority on which to make decisions and evaluate claims (Kegan and Lahey, 2009, p. 17). In contrast to someone in the third order, she will not feel torn when facing the contradictions that are bound to arise when interacting with members of and discussing the tenets of other political ideologies.

A politician at the fifth order of mind operates within a self-authored political belief system, one that is comparable to that developed while in the fourth order. The crucial difference, however, is that he is now able to see the limits of this system, while his fourth-order counterpart could not. Similarly, the fifth-order comparative politician filters the teachings he comes across through the study of other political viewpoints, as he did while in the fourth order, but now he is able to perceive the filter itself as Object (Kegan and Lahey, 2009, p. 19). In other words, he is aware of his filter, can view it as separate from his belief system, and as a result can assess the limitations and constrictions it places on his system as a whole. Furthermore, just as he is able to see the limits of his own system, so too is he able to appreciate the limits inherent to other systems, including those that are present in other political parties. In doing so, he formulates a “trans-ideological” (Kegan, 1994, p. 315) view of different political domains, and rather than take one political viewpoint to be correct and another as incorrect, he is able to gain a deeper understanding of both the costs and benefits inherent in the adherence to each one.

Therefore, as an individual progresses through the different orders of mind, concepts that were once in the subconscious transition to the conscious, and this in turn enables an evolution in the way that individual perceives themselves and their relation to others. While this theory of adult cognitive development does not aim to characterize or describe disease states, it is reasonable to assume that a lack of progression from one stage to the next, or in other words a deficiency in the elements required to move various perceptions and notions from Subject to Object, can contribute to psychological distress and potentially psychiatric disease. This hypothesis will be explored through concepts outlined in the Research Domain Criteria.

## CDT and the research domain criteria

Psychiatric disorders have traditionally been classified based on symptom clusters and clinical phenotypes using the Diagnostic and Statistical Manual of Mental Disorders. However, an initiative pioneered by the National Institutes of Health aims to incorporate multiple methodologies in psychiatric nosology, including genetics, molecular biology, physiology, neural circuitry, and behavior. The Research Domain Criteria, or RDoC, is a framework aimed at using modern research to create a novel taxonomy for mental disorders, and contains five principal domains: negative valence systems, positive valence systems, cognitive systems, systems for social processes, and arousal/modulatory systems (Morris and Cuthbert, 2012).

Within the RDoC framework, CDT most logically falls under the domain of social processes. As can be ascertained from the above example of a political person of different orders of mind, CDT argues that much of the meaning we make of our reality depends upon our interactions with others, both in our immediate circles of family and friends as well as society as a whole. Therefore, within the overall systems for social processes domain, CDT would lie specifically within the subcategories of the *perception of self* and the *perception of others*. To this end, the RDoC serves as a formalized structure for integrating the psychosocial aspects of development and cognition, within which to test various hypotheses and explore different ideas.

Because adult developmental theory currently lies largely in the realm of psychology, making the leap toward the neuroscientific realm with imaging and electrophysiological data must be undertaken gradually. Thus, we shall examine two key concepts relating to these posited developmental changes that already have a well-established basis in neuroscience: Self-related processing and Theory of mind.

## Self-related processing

Having conscious experiences is a large part of what makes us human, and the study of self-awareness is a crucial component of social cognitive neuroscience. Over the past two decades, several functional neuroimaging studies have aimed at delineating the regions of the brain responsible for self-awareness and conscious experience. Cortical midline structures (CMS), including the ventral and dorsal medial prefrontal cortex, as well as the anterior and posterior cingulate cortex have repeatedly shown involvement during tasks of self-assessment (see meta-analysis: Northoff et al., 2006). These tasks range from simple, such as recognizing one's own body or body parts (Thirioux et al., 2010), to more complex, such as making judgments about one's internal emotional state or abilities (Gusnard et al., 2001; Fossati et al., 2003; Schneider et al., 2008; Yaoi et al., 2009; Yoshimura et al., 2009).

The study of mindfulness is also important to consider, as various meditative practices are aimed at bringing awareness to the self. Mindfulness meditation, of which there are many different types, can result in structural changes in the brain, such as increasing the cortical thickness of regions like the prefrontal cortex and insula (Lazar et al., 2005; Santarnecchi et al., 2014; Engen et al., 2017). Neurophysiologic changes as measured through electroencephalography have also been shown to occur as meditators bring awareness to themselves, with distinct differences in EEG profiles depending on experience. For example, a study on Satyananda Yoga practitioners demonstrated that intermediate practitioners with a mean experience of 4 years had increased low frequency oscillations (theta and alpha) in the right superior frontal, right inferior frontal and right anterior temporal lobes, whereas advanced practitioners with a mean experience of 30 years had increased high frequency oscillations (beta and gamma) in the same regions (Thomas et al., 2014).

As argued by Musholt (2013a), it is important to distinguish between having conscious experiences (“being a self”), and being aware of oneself having conscious experiences (“being aware of being a self”). While the former can be a subjective experience, the latter requires an objectification of that experience. To use Kegan's CDT terminology, being a self is considered Subject while being aware of being a self is considered Object. In other words, while it is possible to simply have a subconscious perception of something, it requires a higher level of cognitive development to bring awareness to oneself having that particular perception, or to make Object what was previously Subject. Returning to the previous political example, it would require a higher level of cognitive development to bring awareness to holding a particular political viewpoint, as compared to simply holding that viewpoint and being subject to it.

Studies looking at distinguishing between these two concepts are lacking, mainly because the phenomenology used in the investigation of self-related processing is often indistinct (Legrand, 2007; Flores-González, 2008; Musholt, 2013b). Also, the above referenced studies require some degree of objective awareness of self in order for subjects to participate in the tasks, meaning the tasks themselves contain elements of both subjective and objective self-knowledge and assessment.

In addition, because psychological tasks that assess self-relatedness involve other cognitive processes such as reasoning and memory, it is likely that the brain structures and neural networks involved in self-related processing are common to several cognitive methods. In fact, Legrand and Ruby (2009) go so far as to propose that standard methods targeting the self are in fact non-specific, and any neural activity elicited during participation in self-evaluation tasks can be reduced simply to inferential processing and memory recall. Therefore, in order to properly assess the neural representation of self-awareness, it will be important to develop tasks that are not only specific to processing of the self, but also that attempt to distinguish between the components of Subject and Object therein. Regardless, neuroimaging research in this field has led to inferences that may prove useful in the investigation and treatment of psychiatric disorders such as depression.

### Implications in depression

Rumination is a common characteristic of depressive disorders, in that patients often have an increased tendency toward self-focus, self-assessment and self-appraisal. The nature of that self-related processing, however, tends to be biased toward negative emotional processing. When mapped using neuroimaging, these thoughts localize to anterior cortical midline structures such as the cingulate gyrus (Nejad et al., 2013; Wagner et al., 2015) and medial prefrontal cortex (Lemogne et al., 2012; Li et al., 2017), with these areas demonstrating increased activity in patients with major depressive disorder. In addition, administration of a single dose of the antidepressant mirtazapine has been shown to attenuate this abnormal fMRI activity in these structures (Komulainen et al., 2016), reinforcing their importance in this disorder.

In the field of neuromodulation, these findings have led, in part, to the development of targeted deep brain stimulation (DBS) therapy that is currently under investigation for treatment of severe major depressive disorder. Targeting the anterior cingulate cortex, specifically in the subgenual cingulate area or Brodmann area 25, has shown promising results in the treatment of patients refractory to medications (Mayberg et al., 2005). While only a subset of patients respond to this treatment, further investigation into self-processing networks may shed light on DBS mechanisms in depression, further expanding therapeutic options.

## Theory of mind

Self-related processing encompasses the subjective possession of mental states and the objective awareness of having those states. Theory of mind, however, goes further by encompassing not only the objective awareness of one's own mental states, but also understanding that others possess mental states that can be different from one's own. This is exceedingly important for normal social interaction, as making sense of and predicting the behaviors of other people are essential components of effective communication (Happé, 2003).

Functional MRI studies have implicated the medial prefrontal cortex and temporoparietal junction as important brain regions in theory of mind. These regions are active during complex interconnected mental concepts such as the representation of another individual's actions, desires, and belief systems, the formulation and judgment of other's perspectives, and the inhibition of actions (McCleery et al., 2011; Gweon et al., 2012; Bowman et al., 2017). Furthermore, the activity of these regions during theory of mind tasks correlates with age, in that ventral medial prefrontal cortex is more active in children, dorsal medial prefrontal cortex is more active in adolescence, and temporoparietal junction is more active in adults (Moriguchi et al., 2007; Blakemore, 2012; Sebastian et al., 2012; Vetter et al., 2014), which may point to a developmental component.

Furthermore, electroencephalography (EEG) has been used to study the association between action processing and theory of mind (Marshall and Meltzoff, 2011; Bowman et al., 2017). Event-related decreases in amplitude of alpha rhythm (8–13 Hz) during voluntary actions, termed mu desynchronization, have been shown to correlate with the degree of action production and internal representation of that action. Bowman et al. (2017) has shown that when mu desynchronization was high, a positive correlation was present between action production (i.e., voluntary hand movement), action perception (relationship to the experience or proficiency in action production), and theory of mind (social reasoning), indicating an integrated underlying neural network. Conversely, when mu desynchronization was low, there was a negative correlation between the action and the internal representation, which may be explained by the absence of neural integration for those specific actions. Therefore, the relationship between the neurophysiologic changes during these actions and theory of mind, as measured using scalp EEG, appears to span neural networks.

It is important to determine whether the neural networks and cognitive mechanisms that are used to attribute thoughts and feelings to others are the same as those used to attribute mental states to the self (Happé, 2003). In order for a particular thought or feeling to be attributed to oneself or to another, it must first be recognized *as* a thought or feeling, which means it must be brought from Subject to Object. As expected, this would involve the same neural mechanisms as those in self-related processing, namely the CMS. The mechanisms involved in making Object another person's thoughts or feelings, however, may lie in mirror systems, where certain regions of the brain become active when observing or mimicking the actions of another individual. These regions, which include the inferior frontal gyrus and inferior parietal lobule (Iacoboni and Dapretto, 2006), have been shown in both primates and humans to become active when observing the mental states of others (Gallese, 2007), potentially by enabling direct mapping of another's goals and intentions to the self (Mahy et al., 2014). The understanding of these processes may prove useful in the investigation of diseases such as autism, where individuals have difficulty in understanding and predicting the mental states of others.

### Implications in autism

Autism is a spectrum disorder characterized largely by deficiencies in social interaction and impaired development of theory of mind. A large fMRI study comparing over 400 subjects with autism to normal controls has shown that there is reduced connectivity between brain regions implicated in theory of mind in autistic people (Cheng et al., 2015). Specifically, there was reduced connectivity between areas of facial-expression processing, namely the middle temporal gyrus and ventromedial prefrontal cortex, and spatial functions relating self to the environment, namely the precuneus and superior parietal lobule. Also, neurophysiologic correlates have been described relating repetitive behaviors to changes in alpha-band (8–12 Hz) desynchronization (Keehn et al., 2017), and subsequently attempts have been made to correlate these physiologic changes to differences in information processing, cognition, and behavior (Belmonte, 2017). In addition, transcranial direct current stimulation of left dorsolateral prefrontal cortex (Amatachaya et al., 2014, 2015; D'urso et al., 2015) and right posterior parietal cortex (English et al., 2017) led to improvements in clinical autism severity scales measuring functioning, hyperactivity, and noncompliance. This supports the theory that there is a potentially reversible dysfunction in the mechanism involved in understanding and computing mental states, although it is unclear whether this dysfunction causes autistic symptoms, manifests as a result of autistic behavior, or both.

One of the reasons individuals with autism have difficulty understanding their own mental states and those of others may be that they have difficulty objectifying their emotions and ideas. In other words, they “lack the cognitive machinery to represent their thoughts and feelings *as* thoughts and feelings.” (Happé, 2003) Or, to use terminology from Kegan's CDT, they lack the cognitive machinery to move their thoughts and feelings from Subject to Object. Therefore, investigation into the neural mechanisms underlying these processes may prove useful in developing and honing novel neuromodulatory approaches in the research and treatment of this disorder. As we shall see, computer modeling may aide this investigation by bridging the gap between psychological observations and neural processes.

## Computational models of cognitive development

The observation that behavior can traverse apparently discontinuous stages during development raises the interesting question what kind of changes in the neural processing substrate are responsible for this phenomenon. Computational modeling has been a valuable tool in this respect when studying child development, as it allows simulating the behavioral consequences of particular modifications of the neural processing “hardware.” It can therefore help bridge the gap between psychological observations and the underlying neuroscience.

Early computational models of child development were based on abstract, rule-based representations. While these models provided interesting insights, there is no straightforward mapping between these model structures and processing elements in the neural tissue. This changed to a certain degree with the advent of connectionist models. These models are based on layers of model “neurons,” which are connected to each other to allow excitatory or inhibitory influences of a neuron's activity on the activity of other neurons. While one should be cautious to think of these model “neurons” as being equivalent to individual neurons in the brain, the processing structure is similar enough to be able to make some inferences about neural processing. The results of several studies suggest that developmental stages can be best accounted for when structural change in the network model is allowed (Westermann et al., 2006; Shultz, 2012, 2015). Typically, the network starts out with a particular structure (number of neurons and arrangement in different layers) and is being trained to perform a particular task. Training means changing the strength of connections between neurons in such a way that the network's ability to perform the task is improved, which corresponds to the learning process in real life. The training progresses until the network reaches a stable, but still imperfect performance, at which point new neurons are added to the network. These neurons are then able to compensate for specific weaknesses in the network's performance. Interestingly, the network is typically not able to achieve the same performance when it is comprised of the same total number of neurons from the very beginning, in which case all neurons participate in a distributed representation of the general problem, without being able to provide the specific performance improvement that results from some neurons being added at a later point in time.

Models of development based on Dynamic Field Theory (DFT), however, have demonstrated that developmental stages can also be observed without necessarily having to change the structure of the model in the sense of adding neurons at a later time point (Schlesinger, 2012; Spencer et al., 2012). These models are based on the assumption that some global network parameter, as opposed to the learning-driven changes of the strength of individual connections that have been discussed earlier, changes during development. Examples of such a change would be an adjustment of the overall resting level of a dynamic field or an overall increase in the strength of locally excitatory interactions and laterally inhibitory interactions. Relatively small changes of such parameters can generate qualitative differences in the field dynamics and therefore account for the observation of developmental stages.

While connectionist modeling and DFT appear to be fundamentally different modeling approaches, Thomas et al. (2009) have argued that, although they emphasize different aspects of the developmental process, many key aspects of the mechanisms that give rise to developmental stages are shared between them. The authors therefore encourage constructive integration of the different approaches. Computational models' success in explaining observations of child cognitive development suggests that similar modeling approaches can link adult cognitive development to changes in neural substrate.

## Conclusions and future directions

Cognition has the potential to evolve over the course of adult life. As more complex ideas arise and varied perceptions come to light, newly discovered concepts present novel ways of approaching conflict and making meaning of everyday interactions. This is possible by bringing awareness to mental constructs that were previously in the subconscious, or making Object that which was previously Subject.

Kegan's CDT provides a solid framework within which to explore these ideas. While it is not the only psychological theory of adult cognitive development, its constructs are logical, its concepts are widely applicable, and it has been validated in business and educational settings. Therefore, it serves as a useful model within which to develop and test various hypotheses. However, its basis in psychology makes it difficult to extrapolate those ideas to neuroscientific applications, and as such we must build upon related concepts in an effort to make the leap from the social sciences to the sciences.

Useful related concepts include self-related processing, mindfulness, and theory of mind. While we have explored some of the fMRI and scalp electroencephalographic characteristics of these concepts, invasive human neural recording studies were not discussed because they are distinctly lacking in the literature. Obtaining invasive human neural data is, naturally, invasive, so we propose that human neural recording and stimulation experiments be conducted in individuals undergoing neurosurgical procedures for other purposes. These procedures include intracranial electrodes implanted for the evaluation of epilepsy, deep brain stimulation electrodes implanted for the treatment of movement and psychiatric disorders, and cortical electrodes used during awake brain surgery to aid in the resection of tumors and seizure foci. For example, epilepsy patients with implanted intracranial electrodes for the evaluation of seizures can perform theory of mind or mindfulness tasks while in the hospital. This poses no additional risk to the patient, while providing valuable information in the form of human local field potentials, single unit recordings, and the effects of micro and macro stimulation on task performance.

While cognitive neuroscientific concepts such as memory are increasingly being studied in this fashion, there is an explicit lack of such experiments in social cognitive neuroscience. This lack of human intracranial neurophysiologic data is a significant hindrance to the development of neuromodulatory applications and neuroprosthetic devices that may ultimately interface with neural mechanisms underlying these cognitive developmental processes. We hypothesize that an increase in these types of studies will lead to the creation of novel applications for currently approved devices, such as deep brain stimulation and responsive neurostimulation.

This hypothesis is based on a similar example, that of the historical study of emotions using human intracranial electrophysiology. Through the use of intracranial recordings in the form of local field potentials or single unit recordings, and the use of macrostimulation to mimic particular feelings, several studies have attempted to map various emotions to specific brain regions and networks (for detailed review see Guillory and Bujarski, 2014). These studies have, in part, laid the foundation for the creation of affective computing, a branch of computer science dealing with the study of human emotion and its application to wearable computer devices and robotic technology (Picard, 1997; el Kaliouby et al., 2006). Social communicative prostheses are showing promise in helping autistic individuals understand the complex nuances of human emotion, and subsequently improve communication and social interaction. In a related manner, we have shown how computational modeling can provide insights into discrete cognitive developmental stages, and how these insights could ultimately aid persons suffering from developmental, psychological, or psychiatric disorders.

Intracranial studies carry certain inherent limitations, such as the use of patients with underlying brain pathologies, a physical restriction in recording sites, and the unpredictable effects of stimulation (David et al., 2010). However, the information obtained from electrodes implanted in the brains of awake humans performing psychological tasks is unmatched in spatial and temporal accuracy, and therefore serves as a powerful tool in understanding social cognitive processes. For example, we have seen with fMRI studies that the temporal-parietal junction has implications in theory of mind. Understanding the electrophysiology of this region during theory of mind tasks, as well as aberrations during pathologic states, may allow for the development of an implantable neuroprosthesis that records local field potentials and delivers a stimulation pulse in response to abnormal recordings. These responsive neurostimulation devices are already approved by the FDA for the treatment of epilepsy (Morrell and RNS System in Epilepsy Study Group, 2011), and therefore this technology could be applied to cognitive disorders such as autism once a greater understanding of the neurophysiology is obtained.

As deficiencies in adult cognitive development have been linked to disorders such as autism and depression, we have seen that bridging the gaps between developmental psychology, neuroscience, and modeling has potential implications for clinical practice. Therefore, as neuromodulation techniques such as deep brain and transcranial stimulation continue to advance, interfacing with these systems may lead to the emergence of novel investigational methods and therapeutic strategies in adults suffering from developmental disorders.

## Author contributions

FG and JD conceptualized the idea; FG, DL, AG, and JD prepared the manuscript and performed edits.

### Conflict of interest statement

The authors declare that the research was conducted in the absence of any commercial or financial relationships that could be construed as a potential conflict of interest.
